# A Comparative Proteomic Analysis of the Soluble Immune Factor Environment of Rectal and Oral Mucosa

**DOI:** 10.1371/journal.pone.0100820

**Published:** 2014-06-30

**Authors:** Laura M. Romas, Klara Hasselrot, Lindsay G. Aboud, Kenzie D. Birse, T. Blake Ball, Kristina Broliden, Adam D. Burgener

**Affiliations:** 1 Department of Medical Microbiology, University of Manitoba, Winnipeg, Canada; 2 Karolinska Institutet, Department of Medicine Solna, Unit of Infectious Diseases, Karolinska University Hospital, Center for Molecular Medicine, Stockholm, Sweden; 3 National Laboratory for HIV Immunology, Public Health Agency of Canada, Winnipeg, Canada; 4 Department of Immunology, University of Manitoba, Winnipeg, Canada; Seattle Biomedical Research Institute, United States of America

## Abstract

**Objective:**

Sexual transmission of HIV occurs across a mucosal surface, which contains many soluble immune factors important for HIV immunity. Although the composition of mucosal fluids in the vaginal and oral compartments has been studied extensively, the knowledge of the expression of these factors in the rectal mucosa has been understudied and is very limited. This has particular relevance given that the highest rates of HIV acquisition occur via the rectal tract. To further our understanding of rectal mucosa, this study uses a proteomics approach to characterize immune factor components of rectal fluid, using saliva as a comparison, and evaluates its antiviral activity against HIV.

**Methods:**

Paired salivary fluid (n = 10) and rectal lavage fluid (n = 10) samples were collected from healthy, HIV seronegative individuals. Samples were analyzed by label-free tandem mass spectrometry to comprehensively identify and quantify mucosal immune protein abundance differences between saliva and rectal fluids. The HIV inhibitory capacity of these fluids was further assessed using a TZM-bl reporter cell line.

**Results:**

Of the 315 proteins identified in rectal lavage fluid, 72 had known immune functions, many of which have described anti-HIV activity, including cathelicidin, serpins, cystatins and antileukoproteinase. The majority of immune factors were similarly expressed between fluids, with only 21 differentially abundant (p<0.05, multiple comparison corrected). Notably, rectal mucosa had a high abundance of mucosal immunoglobulins and antiproteases relative to saliva, Rectal lavage limited HIV infection by 40–50% *in vitro* (p<0.05), which is lower than the potent anti-HIV effect of oral mucosal fluid (70–80% inhibition, p<0.005).

**Conclusions:**

This study reveals that rectal mucosa contains many innate immune factors important for host immunity to HIV and can limit viral replication *in vitro*. This indicates an important role for this fluid as the first line of defense against HIV.

## Introduction

Men who have sex with men (MSM) are one of the highest risk groups for HIV acquisition worldwide and are severely impacted by HIV/AIDS pandemic [1]. HIV acquisition is highest in the receptive MSM partner, who can be exposed at the oral or rectal mucosae during unprotected receptive oral intercourse (UROI) and unprotected receptive anal intercourse (URAI). Infection rate per sexual exposure through UROI is estimated to be 0.00–0.04% while URAI is highest at 1.4% [2]. This heterogeneity in transmission at receptive exposure sites is well observed in the literature [3]–[6], but little is known on the biological factors that influence this phenomenon.

HIV first contacts the mucosal epithelium during sexual exposure, which serves as an immune barrier to infection. The oral cavity consists of multi-layered squamous epithelia, while the rectal mucosa consists only of a single layer of columnar epithelia. The reduced thickness of the rectum leads to a higher risk of trauma to the rectal compartment during intercourse. This can cause abrasions in the epithelia for HIV to enter underlying target cells and is thought to be a major contributor to the relatively high risk of HIV acquisition over the oral cavity. The mucosal fluid that overlies the epithelia also contributes to HIV susceptibility, as it is replete with immune proteins to limit pathogen invasion. Soluble protein factors found in oral mucosa such as immunoglobulins, high CC-chemokine levels and HIV binding inhibitors (RANTES and SLPI) have been found to be important in impeding HIV infection at this exposure site [7]–[10]. Certain factors have also been shown to correlate with reduced susceptibility in HIV-exposed uninfected individuals, and are therefore associated with reduced risk of HIV acquisition. These include elevated CC-chemokines and bPRP2 within the saliva of HESN MSM [11], [12]. As well, HIV-neutralizing salivary IgA has been correlated with HIV protection in resistant individuals, and the potent antiviral activity of IgA has made it an attractive vaccine target [13]–[15]. Mucosal immune proteins within the rectum, including potential anti-HIV factors, have yet to be comprehensively described as they have been at other sites of HIV exposure (Table 1). This represents a significant gap in our knowledge of HIV pathogenesis and is a major barrier to understanding HIV transmission through URAI.

**Table 1:** Selected list of immune proteins having described roles in HIV defense found in mucosal fluids.

Mucosal Protein	Antimicrobial Activity	Proposed anti-HIV mechanism	References
Mucins	Physical entrapment, sequestering and clearing of pathogens	Binding inhibitor, inflammation regulator	[39], [40]
Cathelicidins	Disrupts pathogen cell membrane integrity	Replication inhibitor, may increase HIV infection	[41]–[44]
Thrombospondin	Physical entrapment, sequestering and clearing of pathogens	Binding inhibitor	[5]
MIP1α/β (CCL3/4)	Inflammation	Competitive CCR5 binding inhibitor	[45]
SLPI	Disrupts pathogen cell membrane integrity	Binds HIV-cofactor annexin a2, inflammation regulator; epithelial maintenance	[46]–[49]
Mucosal IgA	Physical entrapment, sequestering and clearing of pathogens	Virus neutralization, prevent Trancytosis	[50]–[52]
Basic Proline Rich Proteins (bPRP)	Soluble bPRPs bind dietary tannins and viruses to facilitate clearing. Adherent bRPR2 may promote bacterial infection.	Binding inhibitor	[9], [53]–[55]
Human Neutrophil (α)Defensins	Disrupts pathogen cell membrane integrity	Binding inhibitor, HαD-4 modulates CXC4 expression in target cells, increases HIV susceptibility with prior bacterial infection	[38], [56]–[58] [59]–[61]
Human β-defensin	Disrupts pathogen cell membrane integrity	Chemotactic activity, HβD-2/-3 modulate CXCR4 expression in target cells	[38], [56] [62]
Lactoferrin	Iron sequestering,disrupts pathogen cell membrane integrity	Fusion Inhibitor	[63]–[65]
Lysozymes	Disrupts pathogen cell membrane integrity	Cell killing, antimicrobial defense	[66], [67]
RANTES (CCL5)	Inflammation	Competitive binding inhibition through CCR5 co-receptor	[45]
Elafin/Trappin-2	Bacteriocidal activity	Inflammation regulator	[68]–[70]
Serpin Antiproteases	Regulation of protease activity, regulate inactivation of host defense factors, regulate inflammation, and promote epithelial maintenance	α-1-antitrypsin (Serpin A1), alters NF-kB signaling to inhibit HIV replication in T cells, inhibits fusion via gp41. α-1-antichymotrypsin (Serpin A3), proteolysis of proteins that increase HIV susceptibility	[71]–[74] [75]–[79]
Cystatin Antiprotease	Compliment activation; antigen presentation; inflammation	Cystatin B inhibits HIV replication via STAT-1 pathway activation in monocyte derived macrophages. Oral cystatins (A) found to have anti-HIV activity *in vitro*	[80]–[83] [10] [17]

An important first step in understanding rectal HIV susceptibility due to mucosal fluid is to describe the soluble immune components of rectal mucosa as well as assess the capacity of this fluid to inhibit HIV infection. Characterization of immune factors in these secretions is imperative for our understanding of the frontline role of mucosae at the portals of entry for HIV, and must be considered in the design of preventative strategies or therapeutics that would limit HIV transmission at these sites. Using a proteomic analysis of mucosal secretions, this study is the first comprehensive proteomic analysis of the mucosal proteins within the rectal compartment, using oral mucosa as a reference, in order to address this gap in knowledge.

## Methods

### Ethics

The ethical committee at Karolinska Institutet has approved this study and all participants gave written, informed consent.

### Sample collection and pooling

Mucosal samples were collected from healthy male participants recruited by an advertisement at a blood donor clinic through the Gay Men's Health Clinic in Stockholm, Sweden (n = 10 salivary fluid; n = 10 rectal lavage). Whole, un-stimulated saliva was collected in 50 ml vials, aliquoted and frozen at −80°C; participants were instructed not to eat or drink two hours preceding. During the same visit, rectal lavage was collected after installing 5 ml of sterile PBS and then aspirating the fluid, which was subsequently filtered to remove debris and immediately frozen at −70°C. Low risk individuals were classified as men who have had 0–1 sexual partners and tested negative for HIV (regular plasma screen), chlamydia (throat, urine and rectum) and gonorrhea (throat, urethra and rectum) [14], [16]. The protein concentration of each sample was determined by BCA assay (Novagen). Equal amounts of protein/samples (10 µg) from each individual were combined to create pooled samples (100 µg) for both saliva and rectal lavage for subsequent assays.

### HIV infection assays

HIV infection assays were performed under previously established conditions [17]. Briefly, the TZM-bl reporter cell line was cultured in DMEM media completed with 10% Fetal Bovine Serum (Hyclone Media) and 5% Penicillin-Streptomycin (Fisher Scientific) and incubated at 37°C and 5% CO_2_ for three days [18]–[22]. The TZM-bl reporter cell line was obtained through the NIH AIDS Research and Reference Reagent Program, Division of AIDS, NIAID, NIH: TSM-bl from Dr. John C. Kappes, Dr. Xiaoyun Wu and Tranzyme Inc. Two days prior to infection, 96 well plates were seeded at 10,000 TZM-bl cells per well at the same culture conditions. Just prior to infection, culture media was removed and cells were incubated with 300 µl whole, sterile filtered (0.2 µM) lavage fluid. Salivary fluid was diluted 1∶2 in PBS and rectal lavage fluid was diluted 1∶1.5 in PBS to retain similar concentrations of protein (39.12–0.31 µg/ml salivary fluid and 40.73–0.32 µg protein/ml rectal lavage). Immediately following, an R5-tropic HIV-1 virus (BaL) was added at an M.O.I. of 0.2 (3.92 µl/well), and incubated with cells for 3 hours. Our assays used an R5-tropic strain of HIV that utilize the CD4+ and CCR5+ co-receptors as these are the major infectious strains found to establish a founder population in mucosal tissues, [23] and more specifically, within mucosal T lymphocytes [24]. Negative control wells, containing only virus, cells and PBS, and a positive control containing 10 µM azidothymidine (AZT), virus, cells and PBS were included. Virus and mucosal fluid were then removed and cells were incubated in complete DMEM. TZM-bl cell cytotoxicity in the presence of mucosal fluids was measured using the CellTiter-Glo Luminescent Cell Viability Assay (Promega) according to manufacturer's instructions. Cell viability was measured by a luciferase reaction that produced a luminescent signal proportional to the amount of ATP produced in culture, which was directly proportional to the number of viable cells in each culture. Experimentally treated TZM-bl cells were screened for viral infection after 72 hours incubation (37°C, 5% CO_2_) according to a β-Gal Screen System (Life Technologies) in relative light units (RLUs). Percent infectivity of experimental wells was calculated relative to the negative control and conditions were compared using a two-tailed t-test (α = 0.05).

### Mass spectrometry analysis

Protein isolation, digestion into peptides, and label-free mass spectrometry analysis was performed as described [12], [25] to identify and quantify host proteins (Methods S1). All proteins identified were annotated by function using the peer-reviewed UniProtKB database (www.uniprot.com), and proteins with a known function in immunity were selected for further analysis. Average mucosal immune protein expression was compared across anatomical sites. The average abundance of each protein was calculated within mucosal pools by mass spectrometry and was converted to fold-difference values relative to the mean expression of that protein across mucosal fluids. Data was normalized to a Gaussian distribution using log_2_ transformation, and normalization was confirmed using normal quantile plots. Log_2_(fold-difference) values were further normalized to average protein content so that all positive values correspond to an heightened relative protein abundance, while all negative values correspond to a lower relative abundance of protein. Differentially abundant proteins were determined with two-tailed, independent t-tests (α = 0.05 corrected for multiple comparisons using the Benjimani-Hochberg method) using GraphPad Prism 6.01 (GraphPad Software Inc., La Jolla, CA) and restricted to those with 2.4 or greater fold-difference (effect size = 1.4) between groups to retain an experimental power of 0.8. A full proteins list is available in the supplemental methods (Table S1).

## Results and Discussion

**Figure 1:**
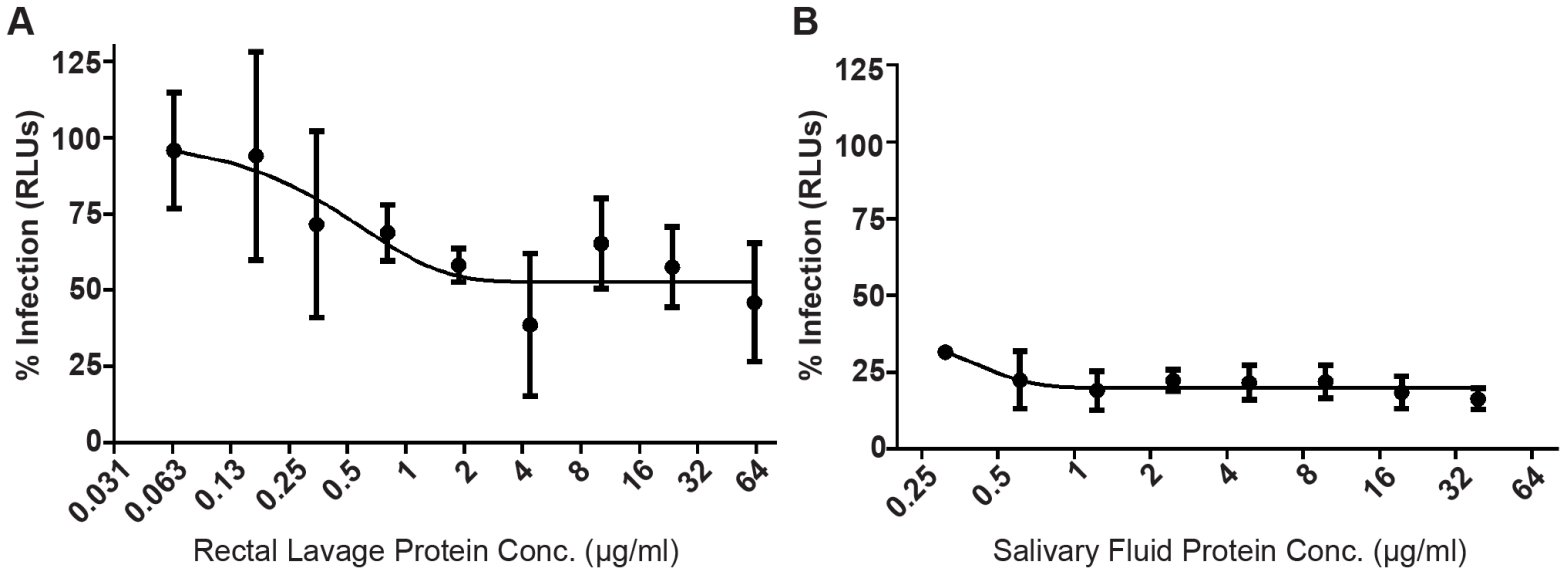
Rectal lavage shows mild inhibitory activity against R5-tropic HIV *in vitro*. Inhibition assays of HIV BaL replication within the CCR5+/CXCR4+ TZM-bl reporter cells in the presence of varying concentrations of rectal lavage and salivary fluid protein were performed. Rectal lavage exhibited a significant, mild inhibitory effect on HIV infection in TZM-bl cells (∼40% inhibition) beginning at 2 µg/ml of protein relative to a negative control (p = 0.05, triplicate assays) (A). Parallel assays demonstrated that salivary fluid had a stronger anti-HIV capacity (∼70–80% inhibition) at as low as 0.3 µg/ml relative to the negative control (p<0.005, triplicate assays) (B). Mucosal fluids were determined to have a negligent effect on cell death based on a luciferase assay that indirectly measured the number of viable cells in each culture via their ATP production (data not shown).

The capacity of rectal mucosal fluid to directly limit HIV replication *in vitro* has, to our knowledge, not been assessed in the literature. HIV inhibition assays were performed by incubating an R5-tropic HIV lab strain (BaL) with TZM-bl reporter cells in the presence of diluted salivary or rectal mucosal fluid. Rectal mucosa demonstrated the ability to inhibit HIV infection by significantly limiting HIV production by approximately 40% at mucosal protein concentrations of 2 µg/µl to a maximum of 61.5% at 64 µg/µl (p = 0.05; Figure 1a). The inhibitory capacity of rectal lavage fluid demonstrated in our assay is relatively mild compared to saliva, which has previously been shown to have a potent effect on HIV infection [26]–[28]. In agreement with the literature, our assays demonstrated that salivary fluid possessed higher antiviral activity, limiting HIV infectivity by 80% at 2 µg/µl (p value = 0.005, Figure 1b). This demonstrates that mucosal fluid can inhibit HIV at physiologically relevant concentrations (6 µg/ml to 68 µg/ml), albeit with lower capacity than saliva. This may have relevance to what is observed *in vivo*, as demonstrated by a much higher incidence of infection upon rectal exposure than through oral exposure [4]. Previously, the low occurrence of HIV orally has been, in part, attributed to high levels of soluble immune factors such as CC-chemokines and the antimicrobial peptides SLPI, LL-37 and defensins [31]. In an attempt to understand the role of these, and other soluble factors in HIV infection at different sites of exposure, we used mass spectrometry to comprehensively define proteins contained within oral and rectal mucosal fluid, and define natural differences within these fluids that may be responsible for the observed discrepancy in HIV inhibition.

**Figure 2:**
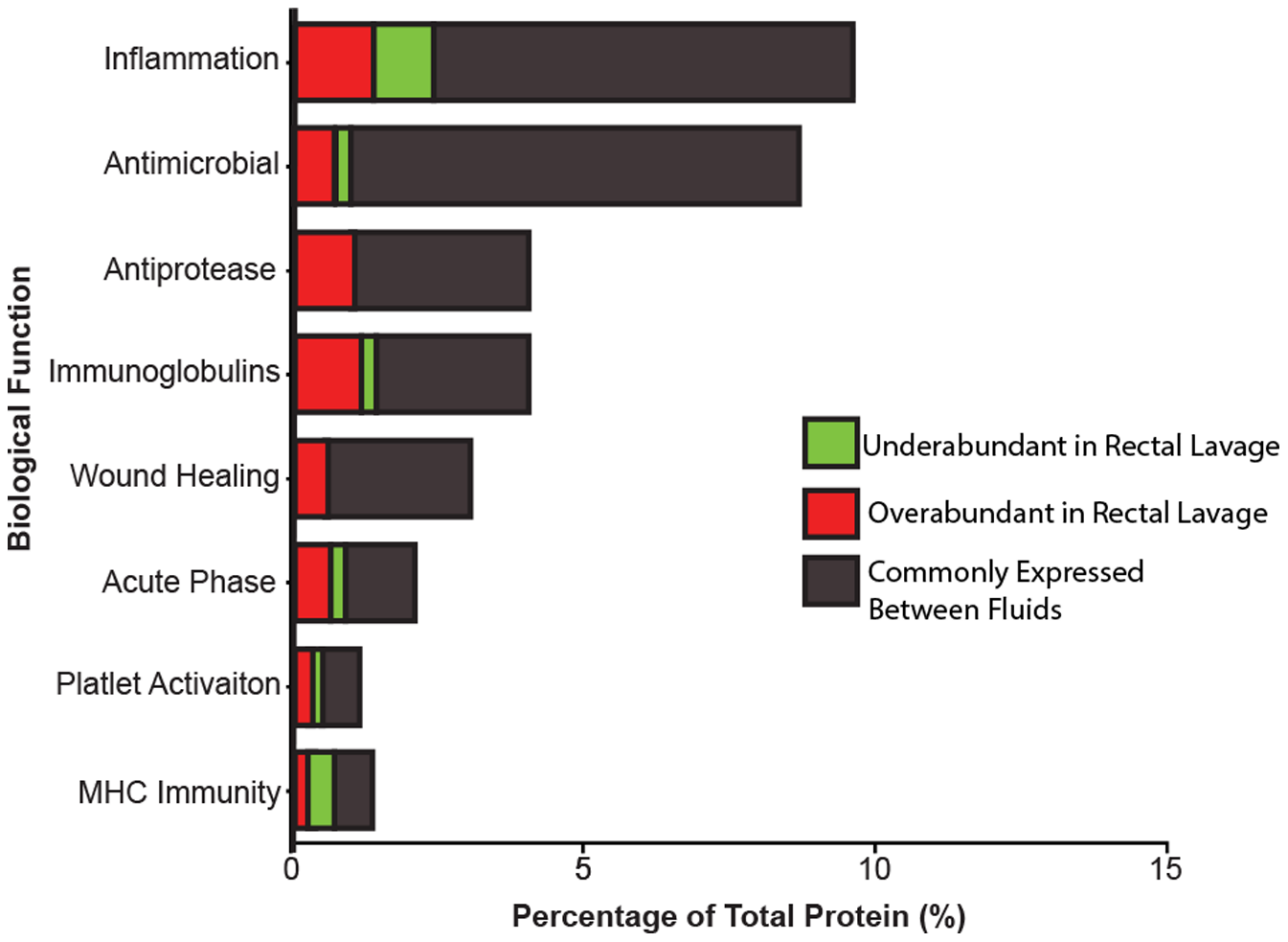
Biological functional categories of immune factors identified in saliva and rectal lavage fluid according to their gene ontology. Proteins identified in both rectal and salivary mucosal fluid pools were annotated by function using the UniprotKB database. Functional analysis found 72 of the 315 identified proteins had functions in immunity. The proportion of the total number of proteins known to possess each given function is displayed (proteins may be found under multiple categories if they have displayed more than one function). Differential expression analysis identified 49 proteins commonly expressed between fluids (grey), 15 overabundant proteins in rectal lavage (red) and 8 proteins underabundant in rectal lavage (green), relative to saliva (p<0.05, corrected for multiple comparisons). Differentially expressed proteins were found in multiple functional categories. The complete list is shown in in Tables 2a and 2b. Functions of proteins that have no known role in immunity are included in Table S1.

Our proteomic analysis identified 315 human proteins expressed in both rectal and salivary mucosal fluid (Table S1). Both fluids contained numerous immune factors with 72 common proteins found to play a role in host defence and immunity (Figure 2). A small portion of proteins were unique to either fluid (one protein was unique to saliva [0.3%] and four proteins were unique to rectal mucosa [1.3%], but none had known roles in immunity (Table S1). The majority of immune proteins identified in both mucosal fluids have known roles in role in inflammation (9.6% of all 315 proteins identified) and/or antimicrobial defence (8.8%; Figure 2). Several other categories of immune proteins were identified within our data set, which included the following: antiproteases (4.0%), immunoglobulins (4.0%), wound healing (3.1%), acute phase response (2.1%), platelet activation (1.2%) and MHC Immunity (1.2%). Within our dataset we identified many proteins without immune function (Table S1); however, our downstream analysis was focused on proteins with known immune function to best determine immunological differences between compartments.

The large majority of proteins identified by mass spectrometry were commonly expressed between the two fluids, with only 29% differentially abundant between saliva and rectal lavage (p<0.05; Tables 2 and 3). However, certain immune factors were found to be higher in abundance in rectal lavage fluid; notably, mucosal immunoglobulins IgA and IgM, known to be important for the binding and clearing of pathogens, increased phagocytosis of microbes and complement activation (Table 2) [29], [30], This may suggest a stronger reliance on immunoglobulin-associated mechanisms of defence in the gut, and supports recent findings on the importance of secreted immunoglobulins in maintaining gut homeostasis [30]. As well, several antiproteases (serpins, inter-alpha trypsin inhibitor, and alpha-2 macroglobulin), known to be important in control of inflammation and tissue remodeling, wound healing proteins (fibronectin) were found overabundant in rectal lavage (Table 2); this may reflect an increased need for the rectal mucosa to control inflammation and repair the thin, damage-prone epithelial layer. Though salivary and rectal mucosal fluid have a similar abundance of most immune proteins, several differentially expressed factors may suggest slightly different immune mechanisms at each mucosal surface that appears to reflect their unique immune requirements. Apart from this trend, the high similarity between these fluids is intuitive when considering that both compartments are a part of the gastrointestinal system.

**Table 2:** Average abundance of proteins significantly overabundant in rectal mucosa relative to salivary fluid as determined by mass spectrometry, and relative expression of these proteins in rectal mucosa compared to saliva.

Protein	Functions	Mean Abundance (x10^3^)	SD Abundance (x10^3^)	Mean Log2 Fold-Difference	SD log2 Fold-Difference	P-value
Calreticulin	MHC Immunity	3855.63	33.87	3.19	0.01	0.001
Protein S100-A7	Antimicrobial,	3459.17	762.25	2.87	0.87	0.006
	Inflammation					
Myosin-reactive Ig k-chain variable region	Ig-mediated immunity	1165.02	189.21	2.78	0.24	0.002
Serpin B3	Antiprotease,	745.43	159.88	2.14	0.32	0.009
	Apoptosis					
Similar to VH-3 family (VH26)D/J protein	Ig-mediated immunity	231.38	25.91	1.66	0.16	0.003
Complement Protein C4-B	Complement Cascade	255.63	52.40	1.35	0.30	0.008
Fibronectin	Wound Healing,	270.48	44.52	1.30	0.24	0.004
	Acute Phase Response					
Inter-alpha-trypsin inhibitor heavy chain H4	Antiprotease,	82.33	6.65	1.28	0.12	0.009
	Acute Phase Response,					
	Inflammation					
Serpin G1	Antiprotease,	669.10	382.28	1.15	0.31	0.01
	Complement Cascade,					
	Acute Phase Response,					
	Wound Healing					
Lambda-chain	Ig-mediated immunity	26045.38	3313.85	1.00	0.18	0.002
Neutrophil gelatinase-associated lipocalin	Antimicrobial	1759.33	72.33	0.90	0.06	0.0006
Apolipoprotein B-100	Inflammation	18.53	1.43	0.87	0.11	0.002
IGA1	Ig-mediated immunity	54074.10	1245.78	0.87	0.03	0.0007
Alpha-2-Macroglobulin	Antiprotease,	1952.70	26.49	0.80	0.02	0.0002
	Complement Cascade,					
	Platelet Degranulation					
IGM	Ig-mediated immunity	2443.25	74.32	0.78	0.04	0.0007

**Table 3:** Average abundance of proteins significantly underabundant in rectal mucosa relative to salivary fluid as determined by mass spectrometry, and relative expression of these proteins in rectal mucosa compared to saliva.

Protein	Functions	Mean Abundance (x10^3^)	SD Abundance (x10^3^)	Mean Log2 Fold-Difference	SD log2 Fold-Difference	P-value
Bactericidal/permeability-increasing protein-like 1	Antimicrobial	3.83	0.816	−2.73	0.31	0.004
Beta-2-microglobulin	MHC Immunity	20.10	0.689	−2.43	0.05	0.00005
Mucin-5B	Antimicrobial	31.85	18.16	−2.00	0.14	0.0006
Interleukin-1 receptor antagonist protein	Acute Phase Response,	2.41	0.099	−1.96	0.06	0.0008
	IL-1 Signaling,					
	Inflammation					
Ig lambda chain V region 4A	Ig-mediated immunity	2.48	0.615	−1.53	0.36	0.008
Zinc-alpha-2-glycoprotein	Antigen Processing	718.00	51.34	−1.33	0.10	0.0009
Metalloproteinase Inhibitor 1	Inflammation	16.24	0.30	−1.11	0.03	0.0001
Moesin	Inflammation	48.94	1.91	−0.49	0.06	0.002

Mass spectrometry analysis found that saliva and rectal mucosa expressed the majority of detected immune factors at similar levels. The oral and rectal mucosae are constantly exposed to food antigens and commensal bacteria, as well as harmful pathogens as a part of the digestive tract; therefore, the oral and rectal mucosal defence systems must be similarly equipped to maintain a defensive barrier against pathogens while avoiding severe immunopathology from constant stimulation [31]–[33]. Both fluids contained defensive pro-inflammatory proteins (complement components and S100 proteins) that promote the activation and recruitment of immune cells, and regulatory anti-inflammatory factors (apolipoproteins) that act to counter inflammation through attenuation of inflammation-signaling pathways (Tables 4 and 5). Mass spectrometry was also able to characterize many proteins with antimicrobial functions outside of inflammatory mechanisms such as pathogen binding/clearing (mucins, deleted in malignant brain tumors 1, peptidoglycan recognition protein) or direct microbicidal activity (lysozyme c, lactoperoxidase and myeloperoxidase); some of these have been found to have specific anti-HIV mechanisms (antileukoproteinase and cathelicidin) and are listed in Table 6. Furthermore, we found most antiproteases (serpins and cystatins) to be commonly expressed between fluids (Table 7). Antiproteases have an emerging role in immune defense at the mucosal surface as they have been found to regulate inflammation, and have been found to be overexpressed in an HIV-exposed yet seronegative (HESN) population of commercial sex workers, implicating a potential role in susceptibility to HIV infection (Table 1). Overall, mass spectrometry was able to provide a novel comprehensive characterization of immune proteins that likely play a role in transmission of HIV across the rectal mucosa.

**Table 4:** Average abundance of ant-inflammatory proteins found in in rectal mucosa as determined by mass spectrometry, and relative expression of these proteins in rectal mucosa compared to saliva.

Protein	Mean Abundance (x10^3^)	SD Abundance (x10^3^)	Mean Log2 Fold-Difference	SD log2 Fold-Difference	P-value
Aminopeptidase N	172.76	191.83	2.13	2.16	0.1
Annexin A1	379.22	88.65	0.81	0.03	0.03
Apolipoprotein A-I	307.73	103.33	0.64	0.49	0.08
Apolipoprotein A-II	16.79	13.08	1.12	1.26	0.1
Apolipoprotein A-IV	152.31	131.37	4.40	1.45	0.01
Apolipoprotein D	126.62	64.73	1.41	0.77	0.05
Apolipoprotein E	43.46	36.21	−0.42	1.38	0.5
Apolipoprotein H	39.91	12.61	0.72	0.46	0.1
Apolipoprotein J	558.80	507.91	1.64	1.56	0.1
CD55	116.85	82.20	−0.12	1.11	0.8
CD59 glycoprotein	43.57	44.97	0.23	1.89	0.8
Glutathione S-transferase P	4.66	1.95	−1.65	0.62	0.02

**Table 5:** Average abundance of pro-inflammatory proteins found in in rectal mucosa as determine by mass spectrometry, and relative expression of these proteins in rectal mucosa compared to saliva.

Protein	Mean Abundance (x10^3^)	SD Abundance (x10^3^)	Mean Log2 Fold-Difference	SD log2 Fold-Difference	P-value
Adenylyl cyclase-associated protein 1	42.81	39.6	−0.22	1.56	0.7
Cathepsin B	416.13	542.07	1.21	3.26	0.4
CD177 antigen	7.67	1.16	−0.07	0.22	0.6
Complement factor C3	87.75	210.03	0.02	0.39	0.9
Complement factor C5	0.27	0.099	−0.11	0.53	0.7
Complement factor C8	0.99	0.69	0.37	1.10	0.5
Complement factor B	771.64	379.74	1.68	0.74	0.03
Complement factor I	5.16	0.94	0.51	0.26	0.1
Fibulin-1	8.59	6.86	0.71	1.24	0.3
Heat shock 20 kDa protein 5	5262.53	7202.34	1.62	4.19	0.4
Heparin cofactor 2	15.05	7.60	3.68	0.76	0.06
IgG	695.54	839.76	0.38	2.59	0.7
Integrin beta-2	1.64	1.80	−1.05	2.11	0.3
Leukotriene A-4 hydrolase	12.71	12.71	−0.15	1.80	0.8
Peroxiredoxin-1	4299.01	6000.28	1.65	5.13	0.5
Phospholipase B-like 1	28.34	12.08	0.99	0.63	0.05
Plasminogen	43.25	16.18	0.93	0.55	0.04
Plastin-2	589.60	93.16	0.68	0.23	0.02
Protein S100-A12	31.00	1.43	−0.29	0.07	0.04
Protein S100-A2	256.25	300.54	1.27	2.42	0.3
Protein S100-A8	478.75	386.65	0.37	1.32	0.5
Protein S100-A9	979.83	705.56	0.47	1.15	0.4
Purine nucleotide phosphorylase	4.19	5.40	1.20	3.12	0.4
Thymidine phosphorylase	248.74	235.85	1.87	1.66	0.09
Ubiquitin C	1704.32	654.21	1.73	0.57	0.02

**Table 6:** Average abundance of antimicrobial proteins found in in rectal mucosa as determine by mass spectrometry, and relative expression of these antimicrobials in rectal mucosa compared to saliva.

Protein	Mean Abundance (x10^3^)	SD Abundance (x10^3^)	Mean Log2 Fold-Difference	SD log2 Fold-Difference	P-value
Annexin A3	100.78	54.84	−0.55	0.83	0.2
Antileukoproteinase (SLPI)	91.02	53.40	−1.44	0.90	0.05
Cathelicidin antimicrobial peptide precursor	53.66	19.67	−0.15	0.54	0.5
Deleted in malignant brain tumors 1 protein	1051.50	1253.13	−1.42	2.51	0.3
Haptoglobin	942.93	160.676	−0.19	0.25	0.2
Lactoperoxidase	83.36	34.97	−1.30	0.62	0.03
Lysozyme C	1283.64	668.15	0.01	0.79	0.7
Mucin-2	73.22	63.81	1.62	1.47	0.09
Mucin-5AC	31.85	18.16	0.56	0.87	0.2
Mucin-7	49.71	38.35	−2.21	1.25	0.04
Myeloperoxidase	1196.32	389.22	0.90	0.48	0.03
Peptidoglycan recognition protein 1	1.67	0.68	−0.98	0.60	0.06

**Table 7:** Average abundance of antiprotease proteins found in in rectal mucosa as determine by mass spectrometry, and relative expression of these proteins in rectal mucosa compared to saliva.

Protein	Mean Abundance (x10^3^)	SD Abundance (x10^3^)	Mean Log2 Fold-Difference	SD log2 Fold-Difference	P-value
Alpha-2-macroglobulin-like protein 1	794.93	235.99	0.64	0.43	0.06
Cystatin-A	29.08	13.28	0.08	0.68	0.8
Cystatin-B	1266.41	1618.59	−0.82	3.04	0.5
Cystatin-C	40.93	13264.45	−0.95	0.48	0.03
Cystatin-D	181.60	212.00	−1.52	2.39	0.2
Cystatin-S	629.27	423.78	−2.64	1.06	0.02
Cystatin-SA	1918.04	1811.97	−0.81	1.65	0.3
Cystatin-SN	159.41	2.08	−0.73	0.02	0.06
Serpin A1	135.86	36.35	0.02	0.39	0.9
Serpin A3	39.55	24.09	0.81	0.94	0.1
Serpin B1	113.39	26.87	0.09	0.35	0.5
Serpin B12	1.46	1.20	−0.56	1.35	0.4
Serpin B13	80.20	54.63	0.87	1.07	0.1
Serpin C1	1.65	0.72	−0.53	0.65	0.2

As this study was an examination of the mucosal proteome of Caucasian men from Sweden it is possible that these findings are restricted to this gender and/or certain populations. Variation between the male and female rectal compartment, as well as variation between populations have been previously described and may influence immune protein expression at each site. For example, current research in women suggests that mucosal factors fluctuate with hormone levels during the menstrual cycle [34], [35]. As the rectal compartment contains hormone receptors, such as luteinizing hormone (LH) receptor, that have been found to fluctuate throughout the menstrual cycle in mammalian rectal tissue [36], it is plausible that sex hormone differences between genders may impact mucosal immunity in the rectal compartment; however, further studies are needed to fully elucidate the role of LH and other sex hormones, such as estrogen, progesterone and testosterone, in mucosal immunity in both men and women to fully determine this impact. As well, the ethnic profile of our population may not apply to other populations for several reasons, including underlying genetic factors or environment. The effect of diet and microflora composition on secreted mucosal immune factors is not fully understood; however, both are known to influence the intestinal immune system [37]. These factors may cause variability in secreted immune factors and result in variation between populations and would be an important consideration for the interpretation of future proteomic data from different cohorts.

Our characterization of rectal mucosa is an important early step in understanding the natural soluble components of this immune barrier. Our HIV infectivity assays demonstrated the capacity of rectal mucosa to inhibit HIV *in vitro*, establishing it as a determinant of HIV infection that warrants future investigation to understand its role in HIV susceptibility. Our findings suggest that, at the level of the proteome, rectal mucosa is equipped with many immune factors known to be important in HIV acquisition through the oral compartment; however, the relatively high abundance of mucosal immunoglobulins and antiproteases in rectal mucosa suggests that its mucosal defense may rely on immunoglobulin-mediated and/or anti-inflammatory immune mechanisms; this could be exploited for HIV vaccine development. The lower inhibition capacity of rectal fluid compared to saliva may contribute to relative susceptibility between compartments to HIV. This difference in inhibitory capacity between fluids may also be due to other factors below the detection threshold of our proteomic analysis. This includes altered levels of CC-chemokines/cytokines which are known to be in high abundance in salivary fluid [7], and other short antimicrobial peptides [38] that are critical factors in HIV infection, and have previously been characterized within this cohort in the context of saliva [11]. Further studies into the HIV inhibitory capacity and soluble immune composition of rectal mucosa from individuals may be warranted to better understand natural variations in HIV susceptibility within populations. Furthermore, investigation into small immune proteins/peptides, as well as secreted factors from commensal bacteria will be necessary to fully understand the soluble immune response within rectal mucosa.

## Conclusions

This comprehensive proteomic analysis of rectal mucosa provides critical information on the immune factor composition of this fluid that may be important for HIV acquisition. Our study includes important information on the rectal proteome in the context of HIV, but highlights several gaps in our knowledge of the subject. High susceptibility in the rectum combined with a paucity of knowledge on HIV transmission dynamics at the rectal mucosa emphasizes the critical need to further investigate this front-line barrier. This research may help in the development of preventative technologies against the rectal transmission of HIV.

## Supporting Information

Methods S1 (DOCX)

Table S1 **A comparative proteomic analysis of the soluble immune factor environment of rectal and oral mucosa: Proteins Identified by Label Free MS/MS in Rectal and Salivary Mucosal Fluid.**
(XLSX)
