# Supplementary material for: A Comparative Proteomic Analysis of the Soluble Immune Factor Environment of Rectal and Oral Mucosa
Source: PLoS One. 2014 Jun 30;9(6):e100820. doi: 10.1371/journal.pone.0100820 (PMC4076261; doi:10.1371/journal.pone.0100820)
Supplement: Methods S1 — (DOCX) [file pone.0100820.s001.docx]

**Supplemental Methods S1: A comparative proteomic analysis of the soluble immune factor environment of rectal and oral mucosa**

***Protein digestion and preparation for mass spectrometry analysis***

All sample types had their protein content measured by standard BCA protein assay (Novagen). Ten micrograms of protein were collected from 10 different individuals to make 100 μg pools for each mucosal sample type. Each pool (rectal lavage or saliva) was treated with urea-exchange buffer (8M Urea, 50mM Hepes, pH 8.0) for 30 minutes at room temperature, and placed into Nanosep filter cartridges (10 kDa). After centrifugation samples were treated with dithiothreitol (25 mM) for 15 minutes, then iodoacetamide (50 mM) for 15 minutes, and washed with ammonium bicarbonate buffer (50 mM). Trypsin (Promega) was added (1.5 µg/100 µg protein) and incubated at 37^o^C overnight in the cartridge. Peptides were eluted off the filter with 50 mM HEPES and were dried via vacuum centrifugation and cleaned of salts and detergents by reversed-phase liquid chromatography (high pH RP, Agilent 1200 series micro-flow pump, Water XBridge column) using a step-function gradient such that all peptides elute into a single fraction for each sample. The fractions were then dried via vacuum centrifugation and kept at -80^o^C and analyzed by label-free mass spectrometry.

***Label-free mass spectrometry analysis***

Fractions were re-suspended in 2% acetonitrile (Fisher Scientific), 0.1% formic acid (EMD Canada) and injected into a nano-flow LC system (Easy nLC, Thermo Fisher) connected inline to a LTQ Orbitrap Velos (Thermo Fisher) mass spectrometer. A 2 cm long, 5 µm particle-sized C18 column ReproSil-Pur C18-AQ resin (Dr. Maisch) was used for peptide trapping and desalting. A 15 cm long, 3 µm particle-sized C18 column (Thermo, West Palm Beach, USA) was used for peptide separation. The elution gradient was from 100% buffer A (2% acetonitrile, 0.1% formic acid) to 32% buffer B (98% acetonitrile, 0.1% formic acid) in 12 minutes at a constant flow of 300 nl/min. MS spectra were acquired on the Orbitrap analyzer at 60000 resolution. After each MS spectrum, and automatic selection of the 10 most intense precursor ions were selected for fragmentation by CID, at 35% normalized collision energy.

***Label-free proteomics data analysis***

Protein identification was performed using Mascot (v2.4, Matrix Science) with IPIHuman database (v3.87) restricting taxonomy to Human. Scaffold (v3.6.4, Proteome Software) was used to validate the protein identifications using the following criteria: 80% confidence for peptide identification, 99% confidence for protein identification, and at least 2 peptides identified per protein. The Scaffold data was imported into Progenesis LC-MS software (v4.0, Nonlinear Dynamics) to perform label-free differential protein expression analysis based on MS peak intensities. Feature detection, normalization, and quantification were all performed using default settings from the software. Retention time alignment was performed using automatic settings, and was manually reviewed for correctness. Only charge states between 2+ and 10+ were included to exclude contaminations from the analysis.
